# Clinical Reasoning: A Missing Piece for Improving Evidence-Based Assessment in Psychology

**DOI:** 10.3390/jintelligence11020026

**Published:** 2023-01-26

**Authors:** Gabrielle Wilcox, Meadow Schroeder, Michelle A. Drefs

**Affiliations:** School and Applied Child Psychology, Werklund School of Education, University of Calgary, Calgary, AB T2N 1N4, Canada

**Keywords:** clinical reasoning, critical thinking, evidence-based assessment

## Abstract

Clinical reasoning is a foundational component of conducting evidence-based psychological assessments. In spite of its importance, limited attention has been paid to the teaching or measurement of clinical reasoning skills relative to psychological assessment, as well as how clinical reasoning develops or how its efficacy can be measured. Improving clinical reasoning throughout the assessment process, from initial case conceptualization to hypotheses testing, to recommendation writing, has the potential to address commonly noted concerns regarding diagnostic accuracy, as well as the accessibility and utility of psychological reports and recommendations, and will, ultimately, lead to improved outcomes for clients. Consequently, we provide a definition of clinical reasoning in relation to psychological assessment, followed by a critique of graduate training assessment and the current challenges of measuring clinical reasoning in psychology. Lastly, this paper provides suggestions for how to incorporate clinical reasoning throughout the assessment process as a way to answer client questions more effectively and provide meaningful recommendations to improve outcomes.

## 1. Introduction

Evidence-based assessment (EBA) is a relatively new concept in psychology that emphasizes the theory and research in selecting and using high-quality assessment methods and processes (Youngstrom and Van Meter 2016). Although there are no agreed-upon standards for its application in psychology, there have been some attempts at providing guidelines for EBA, based on the American Psychological Association’s (American Psychological Association 2006) three recommendations for evidence-based psychological practice, including: (a) using the best available research, (b) applying clinical expertise, and (c) attending to patient characteristics, culture, and preferences (Bornstein 2017). Others have noted that EBA requires effective critical thinking and reasoning, which informs all aspects of assessment, from determining the questions and choosing assessment measures to interpreting the results by analyzing information and data within the context of a client (Dombrowski et al. 2021; Victor-Chmil 2013; Ward 2019). Thus, clinical reasoning supports clinicians who must engage in clinical reasoning during assessment and make diagnostic decisions when presenting client problems in EBA.

The purpose of this paper is to describe the current state of clinical reasoning research in the context of psychological assessment and to propose potential directions for promoting clinical reasoning in assessment practice. This paper will first define the role of clinical reasoning in evidence-based assessment and the research related to this area, outlining some of the contemporary challenges in the training and research related to clinical reasoning in assessment. The second section will summarize the current, albeit limited, literature on how psychologists develop clinical reasoning skills, along with recommendations for extending the research findings on deliberate practice (DP). Finally, this paper will suggest how practitioners might be able to improve their clinical reasoning in assessment contexts, based on the findings of medicine and psychotherapy.

## 2. The Role of Clinical Reasoning in Evidence-Based ()Assessment

Victor-Chmil (2013) posited that “*critical thinking* is the cognitive processes used for analyzing knowledge” (p. 34) and also that “*clinical reasoning* is the cognitive and metacognitive processes for analyzing knowledge relative to a clinical situation or specific patient” (Victor-Chmil 2013, p. 34). Often used interchangeably with other terms such as critical reasoning, clinical reasoning allows psychologists to make sense of a large amount of data as they develop working hypotheses, identify information that supports or refutes those hypotheses, and compare data to diagnostic criteria. Both critical reasoning and clinical reasoning involve intentionally thinking about a problem, testing hypotheses, and generating solutions to the problem (American Psychological Association n.d.; Gruppen 2017). Critical thinking requires not only attending to the outcome of the process but also attending to the process of thinking, which is often omitted in research on assessment (Gambrill 2019). Because clinical reasoning and critical reasoning are notoriously poorly or inconsistently defined within the literature, and because there is considerable overlap between these two terms, they are considered similar enough that we have used clinical reasoning in this paper, due to its more common use within the broader research literature.

In order to move toward EBA and utilize clinical expertise in this process, it is important to understand the current challenges of implementing EBA (Ward 2019). One challenge is in understanding how clinicians gain and apply the foundational skill of clinical reasoning in psychological assessment (Dombrowski et al. 2021). Reasoning is an under-discussed topic in EBA (Wright et al. 2022) that is used when testing hypotheses related to clients’ functioning within their context, synthesizing and integrating data from multiple sources, and providing diagnoses and meaningful treatment recommendations to improve functioning (Mash and Hunsley 2005; Wright et al. 2022; Youngstrom et al. 2015; Youngstrom and Van Meter 2016). When performed well, clinical reasoning aids psychologists in asking important questions to ensure that consideration is given to how psychologists’ beliefs about clients or their problems influence the assessment process.

Unfortunately, faulty clinical reasoning can lead to misdiagnoses and may harm clients through delayed, insufficient, or inappropriate treatment, which ultimately leads to a lack of faith in psychological services (Gambrill 2012; Wright 2021). Currently, there are no available statistics on how faulty clinical reasoning affects the general population because of the difficulty in directly connecting error rates in psychology to negative outcomes (Gambrill 2012). This contrasts with the medical field, where there are considerably more publications on this topic owing to the availability within the medical field of more objective measures of error rates, such as mortality and the length of hospital stays (e.g., Ahmed et al. 2015). Specific to psychology, the link between poor critical reasoning and negative client outcomes is largely indirect and has primarily been examined in relation to the common types and sources of errors in both the testing and report-writing processes, largely ignoring the role of critical reasoning in these problems. Because of the important role that psychological assessment can play in improving client functioning, understanding how psychologists think and reason critically throughout the process of assessment and case conceptualization is vital for improving the quality of assessments (Siegert 1999). Additionally, while there have been significant advances in evidence-based treatments, the lack of corresponding attention to EBA is surprising, as treatment selection should be informed by assessment (Mash and Hunsley 2005).

The pursuit of clinical reasoning in assessment is an important goal. The conclusions and diagnostic decisions derived from psychoeducational assessment can have a significant effect on the daily lives of clients. For instance, an understanding of the ecological factors that either support or restrict the success of a student with academic difficulties is critical in determining whether or not the student meets the diagnostic criteria for a learning disability, and identifying the appropriate remediation, learning support at home and school, and accommodations that are specific to that pupil’s educational needs.

## 3. Examining the Current State of Research Training, Research, and Practice

As Gordon et al. (2022) aptly remarked, “Clinical reasoning is a topic that often feels familiar (or even obvious) … [however,] this sense of familiarity may be masking important differences in how it is understood, operationalized, and assessed” (p. 109). Indeed, how psychologists engage in clinical reasoning during assessment has largely been neglected in the literature (Mash and Hunsley 2005). In discussing the current state of clinical reasoning in psychology, we have drawn upon research into the technical aspects of test administration (Oak et al. 2019), the use of base rates (Burns 1990), diagnostic accuracy in assessment (Aspel et al. 1998; de Mesquita 1992; Watkins 2009), and improving report writing (Nelson 2021; Pelco et al. 2009; Postal et al. 2018), but this body of literature had not addressed how to develop and improve critical reasoning in psychological assessment. Much of the argument of this paper is based on the research of clinical reasoning skills in social work (Gambrill 2012, 2019), medicine (Young et al. 2020), and psychological therapy (Miller et al. 2020) because clinical reasoning and how to develop and improve clinical reasoning in psychological assessment has largely been ignored. Below, we review what is known about clinical reasoning from the literature, highlighting issues with how it is taught, researched, and, currently, practiced.

### 3.1. A Focus on Testing Rather than Assessment

One of the challenges in understanding the role of clinical reasoning in assessment has been the commonplace conflation of the terms, “testing” and “assessment”. In training assessment skills, an emphasis on standardized assessment and reducing administrative error in training programs is warranted, as standardized administration requires considerable training, and critical thinking is predicated on quality data. However, paying attention to testing, including choosing appropriate measures with strong psychometric properties and interpreting test scores appropriately, is imperative but it is insufficient to ensure strong clinical reasoning. Testing generally refers to choosing and administrating measures and assessment alignments. Assessment, however, refers to the entire process, from choosing what questions to ask during the initial interview to interpreting all of the data gathered, including but not limited to test scores (Canivez 2019; Suhr 2015; Wright 2021); the initial steps inform the subsequent hypotheses and guide the assessment process, but they occur prior to test selection, administration, and interpretation (Ward 2019). One problem with most evaluations of assessment skills in training is that there is an emphasis on evaluating the psychometric aspects of assessment and standardized test administration, at the expense of clinical reasoning development (Mash and Hunsley 2005; Wright 2021). There is a danger in focusing on the generation of test scores at the expense of clinical reasoning. Psychologists can use psychometrically strong measures and administer them appropriately but will come to poor conclusions if they do not have the clinical reasoning skills to determine what the problem is that is being presented, in order to ask and answer the right questions or to integrate and interpret the resulting data effectively (Mash and Hunsley 2005).

During the psychological assessment process, test scores are an important source of information. Learning the standardized test measures is a complex and time-consuming task that represents an important foundational skill for reducing error and increasing reliability. Error is inherent in testing for various reasons such as client and examiner factors, as well as problematic testing conditions, including incomplete data, time pressures, and complex environments; therefore, it is important to reduce administrative error as much as possible. Unfortunately, despite the focus on a standardized assessment, errors are common. For example, despite the fact that these are learned skills that are a core part of training programs, assessment errors are commonplace, with practitioners often making more errors than students (Oak et al. 2019). This level of difficulty in accurately implementing skills that are essential for assessment contributes to poor clinical reasoning by providing poor-quality data.

### 3.2. Test-by-Test Reporting

The concern that emphasizing test scores over assessment can lead to weak clinical reasoning is demonstrated by the dominant test-by-test approach used in report writing, which some argue reflects the quality of clinical reasoning (Pelco et al. 2009). It is important that reports are transparent when explaining how the psychologist arrived at their diagnostic conclusions, along with how the assessment process informed the diagnostic decision and recommendations, but test-by-test reports do not make psychologists’ reasoning transparent (Pelco et al. 2009; Wilcox and Schroeder 2015). Weak clinical reasoning can contribute to unclear reports that do not support the clients. In this regard, errors in both the assessment and report-writing processes provide indirect evidence of the association between poor clinical reasoning and negative client outcomes.

Along these lines, Wright (2021) has cogently described the current state of clinical reasoning in assessment: “Psychological assessment has long been a mysterious, intuited process, taught to psychologists in training, test by test, with components of conceptualization, integration, and report writing somewhat tacked onto the end of the process” (p. 3). The test-by-test report style remains the most common technique used by psychologists (Pelco et al. 2009), despite being cited as problematic in the literature (Postal et al. 2018). Test-by-test reports can be a symptom of weak clinical reasoning because psychologists do not integrate other sources of information (e.g., observational data, background information) with the test scores in a meaningful way that will tell a story as to why the clients are struggling, along with the strengths that support them. Meyer et al. (2001) provided a clear explanation of the role of tests within an assessment, stating that “[T]ests do not think for themselves, nor do they directly communicate with patients. As in the case of a stethoscope, a blood pressure gauge, or an MRI scan, a psychological test is a dumb tool, and the worth of the tool cannot be separated from the sophistication of the clinician who draws inferences from it and then communicates with patients and professionals” (p. 153).

Clinical reasoning is more than interpreting test scores. Test scores should be connected to other information, including how clients attained their scores, error analysis, observation, and reports from selves and others. These additional data support a clear argument for how the conclusions were made. Assessment should also integrate client characteristics and functioning and the contextual aspects of the client’s strengths and challenges, in order to inform interventions (Wright et al. 2022). Unfortunately, when information is segmented into individual sections, and test scores are reported in isolation, it is unclear to the reader why the client is experiencing difficulties, making it difficult to generate useful recommendations (Wright 2021).

The magnitude of this issue is highlighted in Dailor and Jacob’s (2011) survey of 208 school psychologists. Of the respondents, 37% read a report within the past year that listed the student’s test scores with no accompanying interpretation; 34% read reports that made recommendations that were unsubstantiated by the data, and 26% read computer-generated reports. Such reports are not useful to readers who depend on them to support clients through follow-up intervention. Limiting the reporting of findings to a list of strengths and weaknesses in the form of test scores reduces the role of the psychologist to that of a psychometrist (Wright et al. 2022). Instead, EBA should utilize an iterative hypothesis-testing and decision-making process that requires well-developed clinical reasoning skills (Suhr 2015; Wright et al. 2022).

## 4. How Do Psychologists Gain Clinical Reasoning Skills?

As a primarily invisible process, identifying how clinical reasoning skills develop through training and experience has been a challenge for both researchers and trainers. This might be the reason why programs spend more time assessing trainee proficiency in test administration than time assessing their broader assessment skills. In addition, there seems to be uncertainty about how or when trainees should learn clinical reasoning skills. Even though clinical reasoning is universally viewed as an important competence outcome by training programs (Harding 2007), programs do not necessarily have a systematic approach to instruction. For instance, there is disagreement as to whether this should be taught in coursework or if it should be acquired through applied experiences such as practica and internship placements. The majority of clinics, schools, and neuropsychologists include assessment in their practice (Arocha and Patel 1995), yet a survey of clinical psychology programs found that less than half of the programs indicated that they teach strategies to improve decision-making and clinical judgment (Harding 2007). This is concerning because it is unlikely that clinical reasoning develops independently, without specific training (Harding 2007). Although the dominant view was once that students acquire these skills unconsciously via clinical experience (Wright 2021), there is growing recognition of the need to explicitly instruct and help trainees to develop accurate clinical reasoning.

Pre-doctoral internships also constitute an opportune period for developing clinical reasoning skills; pre-doctoral internships are generally a time to help students address areas of weakness, in order for them to enter the field with beginning levels of competence. Unfortunately, only 40% of APPIC internship sites offered intensive assessment training for interns (Krishnamurthy et al. 2004). Harding (2007) noted that this lack of training leads to significant concerns about practitioners’ clinical reasoning because, without instruction in this area, psychologists are not likely to realize that they need to improve their clinical reasoning, and consequently, do not actively work to improve their clinical reasoning as they gain more experience. This poses a significant obstacle to psychologists’ ability to provide EBA (Cook et al. 2017). As suggested by Gambrill (2012), clinicians are often unaware of the skills that they are lacking without specific feedback. Consequently, the current research suggests that psychologists do not generally receive enough training in clinical reasoning for assessment during their tenure in graduate programs to gain competence in this area.

### 4.1. Gaining and Measuring Clinical Reasoning

One of the issues with how clinical reasoning in assessment is taught (or not taught), is the limited understanding of what differentiates novices from experts and how much experience or what types of experiences are needed for someone to reach an “expert” level of practice. Researchers have struggled to effectively measure how reasoning develops from novice to expert. There has been an assumption that greater experience results in better clinical reasoning. Practitioners who have more experience should make fewer errors in reasoning and be able to identify what information is important and what legitimately contributes to the overall diagnostic picture. To examine this assumption, some researchers have focused on comparing the differences between experts and novices regarding diagnostic accuracy and reasoning processes.

### 4.2. Diagnostic Accuracy

In comparing the rates of diagnostic accuracy between less experienced clinicians and more experienced clinicians, the underlying assumption is that if the diagnosis is accurate, the clinical reasoning that proceeded it should be accurate as well. However, evaluating the accuracy of diagnostic decisions provides no information about *how* clinicians arrive at their conclusions (Siegert 1999). A focus on diagnostic accuracy is similar to an “outcome bias,” which values outcomes over the quality of the process (Gambrill 2012, 2019). It relegates clinical reasoning to a “black box” where testing information enters and diagnostic conclusions exit, but the transformation process (e.g., clinical reasoning) is a mystery (Siegert 1999; Wright 2021).

Similar to the issues discussed earlier with the test-by-test report-writing style, this emphasis on outcome suggests a process that is directed by test scores, which results in minimizing or neglecting the role of the psychologist in taking responsibility for critically interpreting all of the data, not merely the test scores (Siegert 1999). The narrow focus on diagnostic accuracy fails to identify key differences and issues with the questions that psychologists choose to answer, the tools that they use, and the critical reasoning required to make those decisions and integrate and interpret that information to describe client functioning and to make relevant recommendations.

### 4.3. The Role of Expertise in Clinical Reasoning

Without understanding the clinical reasoning required throughout the assessment process, it is difficult to identify which reasoning practices need to be targeted in training to improve diagnostic accuracy (Siegert 1999). In response, a small body of psychology research has studied the quality of clinical reasoning by examining the reasoning *processes* of practitioners. As with diagnostic accuracy, much of the literature has compared the processes of less experienced with more experienced practitioners. Within the broader literature, there are mixed findings regarding the effect of experience on the process of clinical reasoning.

A study of therapists found that expert therapists specializing in cognitive-behavioral and psychodynamic approaches generated more comprehensive and complex case conceptualizations than did both experienced therapists and trainees (Eells et al. 2005). A study by Arocha and Patel (1995) found that when trainees received contradictory information during case conceptualization, they were unsure how to manage it. Rather than adjusting their hypotheses, they tended to either ignore contradictory findings or interpret those findings to fit their initial hypothesis, rather than adjusting their hypothesis (Arocha and Patel 1995). Trainees also rigidly adhered to rules, paying little attention to contextual factors and, consequently, lacked discretionary judgment (Del Mar et al. 2006). Competent psychologists also demonstrated more skill in coping with pressures, having a broader conceptual framework for their planning, and following general standardized procedures.

The relatively sparse corpus of research focused specifically on psychoeducational assessment suggests that experience leads to limited improvements in clinical reasoning (de Mesquita 1992). For example, a study by Aspel et al. (1998) used a case-based approach to examine the process of clinical reasoning during psychoeducational assessment. Less and more experienced practitioners used similar approaches to the cases and did not change their working hypotheses after reviewing four to five categories of information. In another study, de Mesquita (1992) found experienced school psychologists, with varying levels of education, who considered similar types and amounts of information and came to similar conclusions as less experienced school psychologists. These two studies highlight the fact that experience does not automatically result in expertise. Education and experience were generally unrelated to diagnostic accuracy, and there was little difference among groups in terms of the amount and type of information reviewed and the number of diagnoses made.

However, when de Mesquita (1992) evaluated the process of clinical reasoning undertaken by practitioners, there were differences between less and more experienced practitioners. Practitioners with more experience required less time to reach an accurate diagnostic decision than did students. More experienced psychologists also generated fewer hypotheses and favored one hypothesis based on previous case experience. de Mesquita proposed that experience alone was not beneficial; instead, it was how well that knowledge was conceptually organized that led to accuracy and efficient reasoning.

Although experience seems to benefit psychologists in some ways, it is unclear how much experience is needed for someone to reach an expert level of practice, or if most practitioners even reach that level. Experience can support improvement, but it does not automatically lead to expertise. In medicine, Haynes et al. (2002) noted that expertise is not equivalent to experience. *Expertise* should be judged on one’s knowledge of both the quality of the evidence and skill in interpreting that evidence, considering specific patient circumstances (Haynes et al. 2002). Tracey et al. (2014) found that practitioners gained confidence in their abilities along with experience, but their level of confidence did not match their performance. In fact, after gaining initial skills, confidence increased much more rapidly than accuracy, so the practitioners believed that they were more accurate than they actually were (Sanchez and Dunning 2018). Furthermore, confidence reduced their motivation to reflect on their skills, identify areas of weakness, and actively work to improve them (Tracey et al. 2014). Without awareness of their limitations, clinicians were likely to continue to make the same mistakes after ten years of practice that they made in their first year because there was no opportunity for self-correction (Harding 2007; Watkins 2009). This highlights the importance of separating experience and expertise in understanding the role of clinical reasoning in EBA.

In summary, there is still much uncertainty about how experience and training influence the development of clinical reasoning as trainees move from graduate school to independent practice. The current literature suggests that the profession of psychology has approached clinical reasoning development in an ad hoc way. Relying on practical experiences (i.e., practica) for clinical reasoning development without intentional instruction or opportunities for feedback and reflection has the potential for ineffectual habits to become established, overconfidence to develop in practitioners, and little or no growth over time.

## 5. Moving Clinical Reasoning Skills from Novice to Expert

Research demonstrates that gaining expertise requires an intentional effort in learning and applying the component skills (Chow et al. 2015; Ericsson 2018; Miller et al. 2020) rather than acquiring clinical reasoning skills through supervised practice and then continued independent practice, which appears to the primary vehicle for learning clinical reasoning skills in psychology (Gross et al. 2019; Harding 2007; Krishnamurthy et al. 2004). Consequently, these findings suggest that to gain expertise in clinical reasoning, students require direct instruction and DP rather than simply additional experience. Unfortunately, there is currently no reliable model of assessment for clinical reasoning skills, which makes it difficult to determine where students or psychologists need to improve or how to help them to improve (Miller et al. 2020). As a result, the arguments presented in this section are largely based on research from other areas, and additional research is needed to identify how best these findings might apply to psychoeducational assessment.

### Deliberate Practice

A body of research has examined the benefits of DP on expertise development in a variety of fields, including sports, performing arts, and chess (Ericsson 2018). DP requires clearly defining the individual components of the skill to be learned, immediate feedback in performing the skills, repeated practice of the skills, often in solitary settings, and using information from errors to improve performance (Ericsson 2006). In psychology, the outcomes of using DP in assessment have not yet been studied, although it has been successfully applied to psychotherapy practice. The amount of time that psychologists engaged in solitary DP (e.g., reviewing challenging cases, reviewing therapy recordings, writing down reflections and goals) predicted positive client outcomes during psychotherapy (Chow et al. 2015; Clements-Hickman and Reese 2020). It was more influential than other psychologist demographic variables, including experience, education, race, gender, and theoretical orientation. It is important to note that in DP, solitary practice is informed by feedback and coaching (Ericsson 2018; McLeod 2021; Miller et al. 2020). This was the only psychologist activity that predicted client outcomes and demonstrates both the importance of DP and the difference between experience and expertise.

The main components of DP are “(a) individualized learning objectives, (b) use of a coach, (c) feedback, and (d) successive refinement through repetition” (Miller et al. 2020, p. 39). Goal quality is related to performance levels, wherein the weakest performers do not generally engage in goal setting; average performers create goals focused on the desired outcome without setting smaller proximal goals; the highest performers set goals that break down the larger goal into steps that they will take to achieve the final outcome (Ericsson 2018). The research on implementing DP in therapy uses coaching with feedback because coaches are able to see aspects of performance that are often not evident to the psychologist. Beyond the typical requirements of feedback, such as specificity and timeliness, the feedback should focus on improving specific skills rather than on the final product, refining parts of the clinical reasoning process one step at a time, which leads to better performance in the long run (Miller et al. 2020). One challenge with this process, especially for practicing psychologists, is that implementing changes will result in some failures due to the learning process. This requires a willingness to experience short-term failure in order to improve over the long term (Miller et al. 2020). Instead of focusing solely on how to assess, DP would direct attention to developing the psychologists’ clinical reasoning (Miller et al. 2020). This process of DP has not yet been applied to assessment, but its success in therapy suggests that it is worth exploring this process in the context of assessment.

As with other practices, DP requires intentionality. Miller et al. (2020) offer suggestions for incorporating DP, including scheduling time for it, and protecting it by removing other distractions (e.g., emails or booking another meeting during that time). Taking time every week to jot down notes about what was learned through clinical practice, including successes as well as mistakes that were made and what contributed to them, is one example of an intention DP. Research is needed to determine how to effectively incorporate DP into clinical reasoning during assessment because it is an environment providing limited feedback (Lillienfeld and Basterfield 2020; Tracey et al. 2014). One strategy to improve the awareness of accuracy is to record and monitor one’s diagnostic accuracy and utility over time (Kleinmuntz 1990); unfortunately, psychologists rarely receive this type of feedback from their psychological assessments (Mash and Hunsley 2005), and there is generally a low to moderate level of diagnostic agreement between clinicians (Rettew et al. 2009), making it exceedingly difficult for them to implement this strategy. More work is needed to find effective ways for psychologists to elicit feedback that they can use to inform their evaluations of their assessment practices.

One study found that explicitly teaching medical students how to engage in DP increased their planning and the structure of their work, as well as their performance on clinical exams (Duvivier et al. 2011). However, instruction was only as effective as the student’s engagement with the process and required training in the self-assessment of weaknesses. Not surprisingly, students who were more accurate in their self-assessments performed better than students who were less accurate in their self-assessments (Duvivier et al. 2011).

## 6. Recommendations for Improving Clinical Reasoning

The first recommendation for improving clinical reasoning is to seek feedback throughout the assessment process and after the assessment is over. The nature of brief assessment relationships requires that psychologists intentionally and effortfully seek out this feedback (Siegert 1999). As noted in the work on DP in therapy, it is necessary to seek out negative feedback in order to identify areas of growth, which is necessary to improve practice (Miller et al. 2020). Mental health professionals often fail to acknowledge the uncertainty inherent in the assessment process (Gambrill 2012). Uncertainty throughout the process is inevitable because psychologists work under time constraints, using information of varying quality and completeness, but the negative impact of uncertainty is greater when psychologists fail to acknowledge that it exists (Gambrill 2012). As a result, professionals often overestimate their effectiveness, and those who are the most experienced are both the most confident and the least likely to be attentive to learning from their mistakes (Miller et al. 2020). In fact, overconfidence is one of the cognitive biases garnering the most research, making it an important area for psychologists to consider in their practice (Kahneman et al. 2021).

### 6.1. Framing the Assessment

From the outset, psychologists need to create the space and conditions for effective clinical reasoning. Of particular importance is the intentional practice to move away from the narrow framing of a case (e.g., “Does the client have _____ diagnosis?”) because it similarly narrows the hypotheses generated, data collected, and the data that are considered (Gambrill 2012). Heath and Heath (2013) have argued that when individuals hold one hypothesis, all of their “ego” is invested in it, making it more challenging to actively attempt to disprove it or to pay attention to disconfirming information, increasing the likelihood of engaging in confirmation bias. Putting forth a single hypothesis results in that hypothesis representing them as professionals, making it hard to be open to the possibility that their proposed hypothesis is incorrect. In contrast, developing multiple hypotheses allows the professionals’ egos to be spread across the hypotheses, so as to allow their professional egos to be protected should one or more of their hypotheses be disconfirmed. In order to fully consider multiple hypotheses and to acknowledge the uncertainty inherent in assessment, it may be beneficial to ask what would need to be true for each of them to be the correct diagnosis, making sure to consider those hypotheses in which the psychologist does not initially have much confidence (Heath and Heath 2013).

Opening this space from the outset requires psychologists to reflect on their own assumptions about the client, referral question, and their goals versus client goals, in order to take steps to minimize bias and improve clinical reasoning (Gambrill 2019). It is important for psychologists to identify their assumptions about the client or about the presenting problems so that they can work to move beyond asking questions that reflect their beliefs rather than listening to the actual questions the client would like to have answered (Gambrill 2012). Consideration should also be given to noting potentially negative aspects of the process for clients, including the fact that accessing services may still be challenging after receiving a diagnosis and that recommendations generally require time and effort for the clients and their families (Heath and Heath 2013). This process requires strong listening skills and using motivational interviewing principles to better understand what the client wants to know and the changes to which they are committed in their lives (Suarez 2011). Motivational interviewing has the additional benefit that it can be used to increase client participation and their willingness to engage with later recommendations because it involves the psychologist taking the time to understand client goals and their willingness to make changes; it empowers clients to collaboratively engage in the assessment process (Suarez 2011).

### 6.2. Data Collection

Addressing cognitive biases in clinical practice is beyond the scope of this paper (see Gambrill 2012; 2019; Wilcox and Schroeder 2015). However, the most frequently noted strategy to improve clinical reasoning is to intentionally and systematically seek out information that could disprove the hypothesis, which relates to confirmation bias (Kleinmuntz 1990). Confirmation bias is a common contributor to making poor decisions because, when psychologists invest time and energy in pursuing a single hypothesis, they also invest their ego in it, which makes it more difficult to let the hypothesis go if there is disconfirming evidence. Humans are good at convincing themselves that they are collecting data in order to make a decision, when they are actually garnering support for the decision that they have already made (Heath and Heath 2013), making it important to take intentional steps to acknowledge and minimize confirmation bias in practice. Over-collecting data increases confidence without decreasing the objective uncertainty (Gambrill 2012).

Many assessment errors are the result of inattention and distraction during the test administration or the overconfidence that, with experience, psychologists can administer the test with less active engagement (e.g., reading test instructions verbatim; Oak et al. 2019). As noted above, acknowledging that all psychologists, including ourselves, are at risk of errors, rather than engaging in blind spot bias (e.g., “Others make errors, but I don’t”), is the first step to the increasing awareness of errors and in taking steps to reduce them (Gambrill 2012). It is also important to remember that assessment is more than merely testing (Suhr 2015; Wright 2021). Assessment requires choosing measures to answer specific questions related to hypotheses from case conceptualization, actively approaching the data as a detective, attending not only to the psychometric properties of the measures but also attending to contextual and individual factors and the psychology of human behavior, which includes test scores as one source of data among many (Canivez 2019; Suhr 2015; Wright 2021).

### 6.3. Interpretation and Decision-Making

Psychologists face pressure to find answers for clients to support them in their difficulties, which can make psychologists feel as though they have to provide definitive answers. Psychologists, however, should beware of extremely high levels of confidence in predictive accuracy (Kleinmuntz 1990); they should, instead, practice humble acknowledgment of the limitations of the data available and of human judgment. In line with the ideals of Socratic ignorance, also known as Socratic wisdom, we should acknowledge the limits of the certainty of our conclusions because, as Popper (1996) noted, “… in our infinite ignorance, we are all equal” (p. 5). It is important to remember that there is always uncertainty during assessment; failing to acknowledge that uncertainty can increase errors (Gambrill 2012). We should also make sure to attend to contextual factors rather than only focusing on individual factors within the client, such as data from testing (Gambrill 2012). Finally, psychologists should consider documenting their decision-making process at each step, to increase transparency and access to information that could reveal errors, providing the opportunity to learn from them rather than repeat them (Kahneman et al. 2021). Psychologists should consider several questions to ensure that assessment findings are useful for clients, asking themselves: Do these findings and diagnoses help clients to better understand themselves? Do they inform recommendations that the clients are likely to follow? Do these findings make the clients and their families feel empowered (Nelson 2021)?

### 6.4. Considering Base Rates

Base rates represent one available tool to support clinical reasoning and increase diagnostic accuracy. Meehl (1957) argued that psychologists make more accurate decisions when they use base rates, rather than when they use clinical judgment. Consideration of “the relative frequency of phenomena” or of disorders and behaviors in a population (i.e., base rates; Kamphuis and Finn 2002) is important to consider because many psychologists work in clinical settings where almost all clients are presenting with a problem, making it easy to forget what is typical and what is abnormal in a population.

Base rate fallacy or base rate neglect occurs when practitioners do not use base rates when diagnosing; this results in false positives or negatives in the diagnostic decisions (Koehler 1996). Inattention to base rates is more likely to lead to poor decisions when the base rates conflict with other diagnostic information than when the data are in concordance. Koehler (1996) concluded that decision-makers are often accurate in situations with ample data and when these data are in line with base rates. They are, however, more prone to errors when the base rates are very different from their data. Base rate data can also be challenging due to the complexity of comorbidities that clients present with and the lack of operational definitions of the criteria for disorders (Ward 2019).

When base rate data are available, it is often aggregated (i.e., across the population). This provides the benefit of reducing the bias of individual clinics or psychologists (Reynolds 2016), but it may also obscure actual differences in base rates in a clinical setting as normative-based research sometimes hides individual differences, making them less useful for diagnostic purposes (Ward 2019). In order to effectively use base rates, psychologists need to have information that is specific to their type of practice. For example, the base rate of a specific disorder will be very different in a general practice than in a clinic specializing in a specific disorder, and there may be differences based on other demographic data (e.g., sex, geographical region, ethnicity, age (Youngstrom and Van Meter 2016)).

Although clinicians should consider base rates as part of EBA, there are some noted limitations. First, most studies looking at base rate neglect have been conducted in laboratory settings to find errors (Koehler 1996), leading to a limited understanding of the conditions under which base rate neglect occurs in real-life settings. A lack of information about the occurrence in practical settings makes it unclear how often base rate neglect is a problem, suggesting that the problem might be overemphasized in the research (Koehler 1996). Second, there are no clear guidelines or formulas that psychologists can use to apply base-rate information in their practice (Kleinmuntz 1990). Third, during assessments, psychologists not only diagnose but provide information on the client’s strengths and weaknesses, functioning, and prognosis, which cannot be accounted for by base rates (Garb and Schramke 1996). Further, research is needed to elucidate how to effectively incorporate base rates into practice.

### 6.5. Recommendations and Feedback

Building on the previous discussion of DP, psychologists should seek feedback throughout the assessment process and after the assessment is over. The brief nature of the assessment relationship requires that psychologists intentionally and effortfully seek out this feedback (Siegert 1999). As noted in the work on DP in therapy, it is necessary to seek out negative feedback in order to identify areas of growth, which is necessary to improve practice because psychologists are not likely to receive this important feedback as a matter of course (Miller et al. 2020).

Although not yet a common practice connected to psychoeducational assessments, there is a value in later connecting with clients to assist with the evaluation of clinical reasoning skills in relation to improved client functioning. To maximize the client’s uptake of recommendations, one should be transparent in providing clients with evidence for the effectiveness of an assessment and recommendations, so that clients can make informed decisions (Gambrill 2012). Only 5% of clients think that psychologists’ recommendations are helpful (Postal et al. 2018); when there are five recommendations, the clients will follow just over half of them (Elias et al. 2020). Even worse, about a third of clients do not follow any of the recommendations (Elias et al. 2020). Consequently, it is important to consider how psychologists can use clinical reasoning to improve the usability of recommendations. It may be helpful to work with clients to prioritize recommendations with clients and to engage in premortem planning to identify potential barriers, to ensure that they answer meaningful questions (Heath and Heath 2013), asking clients to think ahead, imagining that they did not implement the recommendation, and identifying what might prevent them from implementing the intervention. Then, the practitioner should work with the client to come up with solutions for each of those barriers. Conversely, it is also possible to ask clients to think ahead and pretend that they did implement the recommendation, and to identify what helped them to implement it. Then, we should work with clients to come up with ways to maximize those supports. This process complements motivational interviewing techniques by empowering clients to identify the recommendations that are the most meaningful to them, and encourages them to take an active role in determining the implementation of recommendations (Suarez 2011).

## 7. Conclusions

Clinical reasoning is an integral part of EBA that is currently poorly understood. As a result, there is little information on how psychologists develop clinical reasoning, how to assess the quality of clinical reasoning during an assessment, or how to gain and improve clinical reasoning skills. This has resulted in recommendations related to pieces of the assessment process, such as test administration, base rates, and report writing, without understanding the role of clinical reasoning in ensuring an EBA that supports clients. This paper outlines the current research in the area of clinical reasoning and draws from work in related fields to provide some initial suggestions on how to intentionally attend to clinical reasoning during an assessment. However, more work is needed to better understand the process of clinical reasoning in assessment, in order to determine the best ways to teach, monitor, and improve the clinical reasoning of psychologists during the assessment process.

## Data Availability

Not applicable.
